# A Screen-and-Treat Strategy Targeting Visceral Leishmaniasis in HIV-Infected Individuals in Endemic East African Countries: The Way Forward?

**DOI:** 10.1371/journal.pntd.0003011

**Published:** 2014-08-07

**Authors:** Johan van Griensven, Ermias Diro, Rogelio Lopez-Velez, Koert Ritmeijer, Marleen Boelaert, Ed E. Zijlstra, Asrat Hailu, Lutgarde Lynen

**Affiliations:** 1 Department of Clinical Sciences, Institute of Tropical Medicine, Antwerp, Belgium; 2 Department of Internal Medicine, University of Gondar, Gondar, Ethiopia; 3 Tropical Medicine. Infectious Diseases Department, Ramón y Cajal Hospital, Madrid, Spain; 4 Public Health Department, Médecins Sans Frontières, Amsterdam, The Netherlands; 5 Department of Public Health, Institute of Tropical Medicine, Antwerp, Belgium; 6 Rotterdam Centre for Tropical Medicine, Rotterdam, The Netherlands; 7 School of Medicine, Addis Ababa University, Addis Ababa, Ethiopia; National Institute of Allergy and Infectious Diseases, United States of America

## Abstract

In the wake of the HIV epidemic, visceral leishmaniasis (VL), a disseminated protozoan infection caused by the *Leishmania donovani* complex, has been re-emerging, particularly in North Ethiopia where up to 40% of patients with VL are co-infected with HIV. Management of VL in HIV co-infection is complicated by increased drug toxicity, and high treatment failure and relapse rates with all currently available drugs, despite initiation of antiretroviral treatment. Tackling *L. donovani* infection before disease onset would thus be a logical approach. A screen-and-treat approach targeting latent or the early stage of infection has successfully been implemented in other HIV-associated opportunistic infections. While conceptually attractive in the context of VL–HIV, the basic understanding and evidence underpinning such an approach is currently lacking. Prospective cohort studies will have to be conducted to quantify the risk of VL in different risk groups and across CD4 cell count levels. This will allow developing clinical prognostic tools, integrating clinical, HIV and *Leishmania* infection markers. Interventional studies will be needed to evaluate prophylactic or pre-emptive treatment strategies for those at risk, ideally relying on an oral (combination) regimen. Issues like tolerability, emergence of resistance and drug interactions will require due attention. The need for maintenance therapy will have to be assessed. Based on the risk–benefit data, VL risk cut-offs will have to be identified to target treatment to those most likely to benefit. Such a strategy should be complemented with early initiation of antiretroviral treatment and other strategies to prevent HIV and *Leishmania* infection.

## VL–HIV Co-infection: A Globally Emerging Issue and Major Threat in East Africa

Visceral leishmaniasis (VL), also called kala-azar, is a vector-borne, disseminated protozoan infection caused by the *Leishmania donovani* complex, predominantly affecting tissue macrophages. Untreated, overt disease is universally lethal. The zoonotic form, with dogs as main reservoir, is caused by *Leishmania infantum* and is prevalent in several regions including the Mediterranean basin, Central Asia and South America, with an estimated total annual incidence of 11,000–19,000 cases/year. The anthroponotic form is caused by *L. donovani*. This form is prevalent in the Indian subcontinent and East Africa (mainly Sudan, South Sudan and Ethiopia) with an estimated annual incidence of 190,000–370,000 cases/year [1]. HIV has been identified as one of the emerging challenges for VL control. HIV infection dramatically increases the risk of progression from asymptomatic *Leishmania* infection towards disease (VL) and VL accelerates HIV disease progression [2]–[8]. Whereas HIV has contributed to the re-emergence of VL in Europe in the 90s, the problem of VL–HIV co-infection is now especially marked in some regions of Ethiopia, where up to 40% of patients with VL are co-infected with HIV [9].

Management of VL–HIV co-infection in East Africa remains unsatisfactory [9], [10]. Despite increased availability of antiretroviral treatment (ART) and more effective anti-leishmanial treatment, the case-fatality rate remains high. Up to 50% of patients fail to clear parasites from infected tissues; recurrent relapse is frequent, ultimately leading to treatment unresponsiveness and overwhelming parasite load [2], [9]–[11]. It is clear that once VL is established in HIV-infected individuals—meaning that *L. donovani* infection has evolved to the disease stage—prognosis at the individual level is dire, despite initiation of ART [2].

## Need for an Upstream Approach: From Prevention to Pre-emptive Treatment

Given these prevailing problems, tackling *L. donovani* infection before disease onset is thus a logical approach. Similar approaches have been successful in other HIV-associated opportunistic infections (Table 1). Cryptococcal disease is equally associated with poor outcomes in HIV co-infected individuals. A screen-and-treat strategy is currently recommended by the World Health Organization (WHO), whereby asymptomatic individuals with a low CD4 count are systematically screened. Those testing positive for early cryptococcal infection prior to ART initiation receive pre-emptive treatment with fluconazole [12], [13]. However, this strategy is underpinned by a number of facts. First, cryptococcal antigenemia can be reliably detected, and lateral flow assays (using blood or urine) have been developed [14]. Second, detection of asymptomatic cryptococcal antigenemia precedes overt disease and is an independent risk factor for mortality [13], [15]. Importantly, pre-emptive treatment of asymptomatic cryptococcal antigenemia with fluconazole can prevent clinical progression towards overt disease and reduce mortality [16]–[18]. Fluconazole is widely available within HIV programs in low-income countries, and drug interactions with ARVs have been well studied. Isoniazid prophylactic therapy targeting latent tuberculosis infection is another WHO-recommended preventive strategy (Table 1). While conceptually attractive in the context of VL–HIV co-infection, the basic understanding and evidence underpinning such an approach is currently lacking. Key aspects and associated knowledge gaps relating to such a strategy are discussed in the following sections.

**Table 1:** Two examples of preventive strategies against HIV-associated opportunistic infections recommended by the World Health Organization.

Population to be screened and aim of the strategy	Marker/test	Treatment (WHO recommended)	Comments on evidence-base; perspectives
**Screen-and-treat strategy for cryptococcal infection (WHO rapid advice 2010) ** [**12**]			
Asymptomatic ARV naïve HIV-infected individuals with baseline CD4 counts <100 cells/µL before ART initiation	Latex agglutination test for cryptococcal antigen in serum: technically demanding; cost: 7?17 USD/test	Fluconazole 800 mg/d for 8 weeks followed by fluconazole 200 mg/d until CD4 counts >200 cells/µL	Evidence base relatively limited: Retrospective observational studies on natural evolution of asymptomatic cryptococcal antigenemia and risk of progression (according to CD4 cell counts)
To target early, active cryptococcal infection to prevent progression to overt cryptococcal disease; (meningitis is associated with high mortality)	Prototype dipstick developed (point of care): for serum/urine: cost: 2.5 USD/test	- Widely available in HIV treatment programs (donation program)	*Treatment*: small retrospective/prospective observational studies; no randomized controlled trials; optimal fluconazole dose to be determined; ARV drug interactions & safety relatively well studied
**Isoniazid prophylactic therapy - IPT (WHO guidelines, 2009)** [76]			
HIV-infected individuals, irrespective of ART use, with active tuberculosis (TB) ruled out	Tuberculosis skin test	Isoniazid prophylactic treatment (IPT) 300 mg/d for 6 months; Longer duration if high TB transmission: effect weans off after IPT interruption	Strong evidence base
To prevent reactivation of latent	- Requires patient to return after 72 hours	- Widely available in a TB & HIV programs (low cost)	- Prospective observational studies to identify risk and risk markers
TB infection (and possibly also reinfection)	- IPT indicated if skin test positive		- Interventional studies on IPT- ARV drug interactions & safety relatively well studied
	- If skin test not available, treatment for all		Interferon-γ release assays to target IPT
			Shorter regimens under evaluation

ARV: antiretrovirals; ART: antiretroviral treatment; WHO: World Health Organization.

## Targeting Asymptomatic *L. donovani* Infection in HIV Co-infection

Increasingly, it has been recognized that asymptomatic *Leishmania* infections generally outnumber VL cases [19]. In some VL-endemic East African regions, up to 20%–30% of the population were found to be asymptomatically infected (latent infection) [20]–[24]. However, how to identify asymptomatic infections is not yet well defined [19], [25]. Different markers have been used, including serological methods, parasitological methods, urine antigen tests and the leishmanin skin test (LST). Whether positivity in any of these tests represents a different stage of infection over time or whether it reflects a different parasite–host interaction and has clinical or prognostic implications is not well known (Table 2) [19].

**Table 2:** Direct and indirect markers of *Leishmania* infection and their features.

	Features and clinical/epidemiological significance	Knowledge gaps; operational issues and challenges
*Direct*		
*PCR on peripheral blood*		
	Presumed recent circulation of viable parasites	No detailed studies in HIV-infected patients of kinetics of PCR prior to VL
	Asymptomatic & immunocompetent: no clinical significance; epidemiological marker of VL exposure	Challenging to implement in resource-constrained settings
	Diseased individuals: diagnosis and post-treatment monitoring	- cost; technically demanding; LAMP in development
	Quantitative thresholds associated with risk of disease progression	- ideally: in dipstick format and semi-quantitative
*Microscopy and culture (peripheral blood)*		
	Circulation of (viable) parasites	Few studies in asymptomatic patients, sensitivity probably very low in asymptomatic infection
	Microscopy on peripheral blood (Giemsa staining)	Leucocyte concentration methods to increase sensitivity under exploration
	Conventional culture (several weeks; technically demanding)	Microculture: more sensitive/faster (but not same-day result)
	Microculture methods: culture in microtiter plates, reading with inverted microscope	
*Latex agglutination test (urine antigen)*		
	Renal excretion of parasite antigen	No data on asymptomatic infection: sensitivity possibly low
	Source unclear: peripheral blood? Renal?	Cost ∼2$/test; next generation assay in development: potential for semi-quantitative dip-stick format;
	Semi-quantitative test (minimal equipment required (boiling step))	
*Indirect*		
*Direct agglutination test (DAT); (serology)*		
	Seroconversion: risk factor for VL; epidemiological marker of recent exposure	Feasible in field setting but requires overnight incubation
		Cost ∼2$/test
		Possibly amendable to dip-stick format;
*rK39 ELISA or RDT (serology)*		
	Seroconversion: risk factor for VL; epidemiological marker of recent exposure	No data on asymptomatic infection in HIV
	ELISA titer associated with treatment response/relapse;	Other serological tests not readily available in RCS
		RDT: cost ∼1$/test
*Leishmanin skin test (LST)*		
	Measures delayed hypersensitivity response (reading after 72 hours)	Delayed responses (in general) can be reduced by immunodeficiency (cfr TB); cost ∼2$/test
	Measure of protection against VL	Requires follow-up visit after 72 hours; limited production
	Mainly used for epidemiological purpose (past exposure)	
*Interferon-release assays (IGRAs)*		
	Detects T-cells in peripheral blood reactive to *Leishmania* antigens	Technically demanding but same day result possible, high cost
	Positive response in active disease and asymptomatic infection	Possibly less affected by immunodeficiency (cfr TB)

ELISA: enzyme-linked immunosorbant assay; LAMP: Loop-mediated isothermal amplification; PCR: polymerase chain reaction; RCS: resource-constrained settings; RDT: rapid diagnostic test; TB: tuberculosis; VL: visceral leishmaniasis.

Asymptomatic *Leishmania* infection has, up to now, predominantly been studied from an epidemiological perspective [19]. In immunocompetent individuals, *Leishmania* infection will usually be contained by the immune system, and treatment of asymptomatic cases is currently not recommended [26]. However, the situation might be entirely different in HIV co-infected individuals. This group is at a substantially higher risk of progression to VL and also faces far-reaching clinical consequences of the development of overt VL, triggering a vicious cycle of repeated VL episodes [2].

With the global scaling-up of ART services, there are now large HIV patient populations living in VL-endemic East African regions and enrolled in HIV care, although still often diagnosed with advanced HIV disease. Until ART-induced CD4 cell count recovery has taken place, such patients are probably at a substantial risk of VL, and could benefit from preventive strategies. Current data in immunocompetent individuals indicate that overt disease is preceded by a prolonged period of asymptomatic infection, which can be detected using different markers of *Leishmania* infection [19], [21]. Typically, most cases of VL occur within a year after primary infection (usually within 6–9 months) [21], although this period could possibly be shorter in HIV patients. Alternatively, VL can occur due to reactivation of a previously acquired latent infection following HIV-induced immunosuppression. Conceptually, there is an opportunity for screening strategies, integrating markers of *Leishmania* infection, to capture those at high risk of VL. Ultimately, this could lead to a screen-and-treat strategy for VL in HIV-infected individuals living in VL-endemic areas, whereby the individual's short-term risk of VL is assessed at every clinical encounter. However, several knowledge gaps will need to be addressed before this strategy can be taken forward to implementation in resource-limited VL-endemic areas.

### Developing prognostic clinical tools

The development of clinical tools to predict the risk of VL in HIV-infected individuals would be a prerequisite for an effective screen-and-treat approach. Such prognostic models, relying on simple clinical and laboratory data, have, for instance, been developed to predict the one-year-term mortality in HIV-infected individuals starting ART, with predicted mortality ranging from below 1% to up to 60% [27]. Likewise, VL prognostic clinical tools could be developed, including clinical information, HIV-related laboratory information (CD4 count) and leishmanial markers. In more complex models, immunological markers and other more advanced biomarkers could be integrated.

#### Direct leishmanial markers

Leishmanial markers either directly detect the parasite or its components, or indirectly detect the immunological reaction against the parasite (Table 2). Amongst the direct markers, molecular tests like the polymerase chain reaction (PCR) are increasingly used. PCR positivity for *Leishmania* on peripheral blood, probably indicating recent circulation of viable parasites, has been found very commonly in VL-endemic regions [19], [20], [28]. The kinetics of PCR-positivity in relation to asymptomatic *Leishmania* infection has, however, not been studied in detail in the immunocompetent nor immunodeficient population. Whether this is short-lived or chronic/intermittent with fluctuating levels and whether this has prognostic implications is not yet well-defined [29]–[31].

PCR (on bone marrow and peripheral blood) has been used as a tool to diagnose VL, monitor treatment response and predict relapse in HIV-infected individuals [32], [33]. With quantitative PCR, thresholds could be identified, predicting the onset of symptoms and progression to disease (VL relapse). However, its value as a screening tool for asymptomatic *Leishmania* infection in HIV-infected individuals has not been evaluated so far. A single study in Brazil (where *L. infantum* is prevalent) found a high prevalence of PCR-positivity in HIV patients enrolled in HIV care. The risk of progression to VL in a population with good access to ART appeared to be low [34]. However, this could be entirely different with *L. donovani* and in populations where access to HIV care is delayed, and co-infections and malnutrition are common. Possibly, repeated positivity or increasing PCR levels in an HIV-infected individual could herald the onset of VL. A (semi-quantitative) dipstick format with different thresholds would be required for field implementation.

Commercially available latex agglutination tests to detect *Leishmania* urine antigen have been explored in Europe in HIV-infected individuals with VL (Table 2) [35]. Whereas repeated antigen positivity was predictive of subsequent relapse, its value as a prognostic marker to predict the onset of VL (i.e., prior to the onset of VL) remains to be determined. For diagnostic purposes, it was found highly specific but insufficiently sensitive, including in East Africa [36]. However, improved tests are in development. The recent development of point-of-care urine antigen tests for tuberculosis and cryptococcal infection can serve as inspiring examples [14], [37].

Other methods, like microscopy or conventional culture on peripheral blood, are unlikely to be sufficiently sensitive in asymptomatic infections in the current format [38], [39]. Micro-culture methods on peripheral blood might possibly be more sensitive [38], [40].

#### Humoral and cellular immune response markers

Amongst the different diagnostic serological tests, the direct agglutination test (DAT) and rK39 rapid diagnostic test (RDT), or ELISA, are most commonly used in resource-limited settings (Table 2). DAT sero-conversion has been identified as a risk factor for VL in the Indian subcontinent and in East Africa [21], [41]. In a recent study, high titer DAT converters had a 100-fold increased risk of VL, reaching up to 12% [42]. Serological responses in asymptomatic infection can be transient and sero-reversion can occur [41]. Of interest, in an Ethiopian study where six-monthly DAT testing was done, recent sero-converters comprised over 90% of VL cases, with around 10% of recent sero-converters subsequently progressing to VL [21]. There are, however, no studies from East Africa specifically focussing on HIV-infected individuals.

There is some evidence supporting rK39 ELISA as a prognostic marker for VL in immunocompetent individuals in Brazil [43] and India [44]. In the latter study, positivity on rK39 testing in contacts of VL cases was found to have predictive values of 44% and 69% for the occurrence of VL over the next 3 and 12 months respectively. There are, however, indications that the antibody response is attenuated in HIV-infected individuals [45]. The antibody response in HIV-infected patients might also depend on whether *Leishmania* infection precedes HIV infection or vice versa. A few small studies from Europe nevertheless provide support for serology—including DAT—as a marker predictive of VL in HIV patients [46], [47].

The LST has been mostly used for epidemiological purposes (Table 2) [21], [23], [24], [48]. A positive LST has consistently been shown to be a reliable indicator of protective immunity [21], [48]–[50]. How HIV infection impacts LST positivity has not been well studied. Similarly, as for tuberculosis, prototype interferon-γ release assays have been developed recently [51]. Their exact value to detect asymptomatic *Leishmania* infection, especially in immunocompromised individuals, remains to be determined.

Besides *Leishmania*-related factors, HIV infection markers (e.g., CD4 count, inflammation markers, HIV-1 viral load) should be evaluated as well. Several cytokines have also been explored as prognostic markers, and a range of other immunological markers (e.g., macrophage activation markers) merit consideration [52]–[60]. Amongst the cytokines, interleukin-10 looks most promising but has not been studied specifically in HIV-infected individuals [61]. Broad-based genetic analysis (post-genomic methods) might provide new leads [62], [63]. For instance for tuberculosis, this has allowed discrimination (“biosignatures”) of uninfected versus latently infected versus diseased individuals [64], [65]. Although such complex methodologies should initially be evaluated in research environments, promising leads might subsequently be amenable to simplification and adaptation to resource-constrained settings [65].

### Drugs for preventive therapy

Choices will have to be made regarding which drugs to assess in clinical VL prevention studies, which may obviously vary across settings and regions. All main drugs currently used to treat VL have a number of limitations (Table 3) [1]. In areas with zoonotic transmission, parenteral administration (every 3–4 weeks) of pentavalent antimonials, lipid formulations of amphotericin B or pentamidine has been recommended for secondary prophylaxis after patent VL in patients at risk of relapse [26], [66].

**Table 3:** The main drugs currently used for treatment of visceral leishmaniasis.

Drug	Regimen	Toxicity	Cost/course^a^	Main issues
**Pentavalent Antimonials**	20 mg/Sb5+/kg iv or im daily for 28–30 days	Frequent, potentially severe; Pancreatitis; Cardio, nephro hepatotoxicity	Generic ∼$53 Branded $70	Length of treatment Painful injection Toxicity: high mortality in co-infected African Patients Resistance in India
**Conventional Amphotericin B**	0.75–1 mg/kg iv for 15–20 doses (daily or alternate days)	Frequent Infusion-related Reactions, Nephrotoxicity	Generic price: ∼$21	Lengthy hospitalisation (in-patient care) Need for slow iv infusion Toxicity Heat stability
**Liposomal Amphotericin B**	3–5 mg/kg/d iv up to total dose of 10–30 mg/kg Single dose (10 mg/kg) in India	Uncommon and mild; Nephrotoxicity (limited)	Preferential price: $280 (20 mg/kg total dose) Commercial price: ∼10×	Price Slow iv infusion Heat stability (<25°C) Single dose not effective in East Africa
**Miltefosine**	Orally daily over 28 days; dose according to age and body weight	Common, usually mild and transient; gastro-intestinal, Nephro + Hepatotoxicity Possibly teratogenic	Preferential price: ∼$74 Commercial price: ∼$150	Price Possibly teratogenic Potential for resistance^b^ Patient compliance Relatively limited efficacy data in East Africa
**Paromomycin Sulphate**	15 mg/kg im daily for 21 Days (Indian subcontinent)	Uncommon, Nephrotoxicity Ototoxicity Hepatotoxicity	∼$15	Efficacy variable between and within regions (less in Sudan) Resistance readily obtained in lab isolates

a Actual costs of the drugs, costs related to the logistics of storage and distribution are not taken into consideration.

b Due to long half-life + low genetic barrier (resistance readily obtained in lab isolates).

iv: intravenous; im: intramuscular.

Ideally, drugs that currently form the backbone of VL treatment programs should not be used for preventive therapy in anthroponotic VL regions (East Africa and the Indian subcontinent), given the potential risk of emergence and spread of drug resistant parasites [24]. However, this argument is relative, since avoiding treatment of multiple poorly responsive VL episodes might also constitute a resistance prevention strategy. Monthly intravenous administration of pentamidine—not a first line drug—is currently under evaluation in Ethiopia (NCT01360762) for individuals at high risk of VL relapse (i.e., secondary prophylaxis). However, it is unclear whether VL preventive strategies relying on repeated parenteral administration of anti-leishmanial drugs will look favourable in terms of cost and risk benefit. Most likely, the risk of progression to VL amongst those likely to benefit from preventive strategies will be lower than the risk of VL relapse (up to 60% by one year) once VL has been established.

Ideally, preventive therapy should be oral, well tolerated, and cheap, similarly as fluconazole for cryptococcal disease, isoniazid for tuberculosis, or co-trimoxazole for toxoplasmosis. Combination therapy might be indicated to shorten treatment and lessen the risk of emergence of drug resistance, or to enhance the efficacy of two moderately active oral drugs [67]. Miltefosine is the only oral drug available, but has substantial gastro-intestinal intolerance, and adherence is often poor (Table 3). Its long half-life and low genetic barrier facilitate the emergence of drug resistance [1]. Miltefosine has been successfully used as maintenance therapy in HIV-infected individuals, in monotherapy, or combined with itraconazole [68]. Fluconazole (combined with allopurinol) and itraconazole were also effective as maintenance therapy in a few case series of immunosuppressed patients [69]–[71].

A number of novel oral drugs are in development. Imipramine, a commonly used oral drug against depression, is readily available, well tolerated, and highly effective in animal models [72]. Similar drugs (e.g., amitriptyline) are often used in HIV care programs in low-income countries to treat neuropathic pain and depression. Such drugs could also be taken for an extended duration, as preventive treatment. Data on clinical efficacy against VL are currently lacking. Fexinidazole is undergoing clinical evaluation [73]. Several other new chemical entities are in development by the Drugs for Neglected Diseases initiative and others [74].

In Europe, there have been indications that parasites found during asymptomatic infection differ from those found during disease [75]. Moreover, parasites causing VL in HIV-infected individuals have been found to be more diverse, with higher enzymatic polymorphism, compared to the general population [2]. There are currently no data whether parasites causing latent infection are susceptible and accessible to anti-leishmanial drugs, let alone in HIV co-infected individuals in East Africa.

### Treatment strategies

In line with other opportunistic infections, preventive treatment strategies could be broadly divided in two categories [66].

Primary prophylaxis (prophylactic therapy) would mainly aim at preventing *Leishmania* infection, or target latent infection, perhaps as indicated by sero-conversion of *Leishmania* serological tests. Such a strategy could possibly be of value with moderate immunosuppression (e.g., CD4 counts of 200–350 cells/µL). Isoniazid preventive therapy targeting latent tuberculosis infection would be the prototype example (Table 1) [76]. A CD4-guided approach, as used with co-trimoxazole primary prophylaxis, would be an alternative simple approach [77]. Such a strategy would particularly be indicated if progression from asymptomatic infection to disease would be found to be too short to be captured during screening.

Pre-emptive therapy would essentially aim at blocking VL progression at an early stage, following detection of parasite replication (e.g., as evidenced by detection of urine antigen and/or a specific pattern or level of PCR positivity) prior to symptom onset. Possibly, this strategy might be indicated for severe immunosuppression (e.g., CD4 counts <100 cells/µL). Screening for cryptococcal antigen in asymptomatic HIV-infected patients at risk (low CD4 cell counts), followed by pre-emptive therapy to block progression to cryptococcal meningitis is a well-known example [13].

A short course of prophylactic or pre-emptive therapy could be effective under the assumption that all parasites can be cleared from the body. In light of the prevailing dogma that sterile cure is not achievable with treatment, this scenario is unlikely [78]. A more likely outcome is that the parasite load would be reduced significantly (“pulse therapy”), so as to reduce the short-term risk of VL. In this scenario, maintenance therapy or repeated treatment cycles might still be indicated to suppress parasite replication until sufficient ART-induced immune recovery has taken place. This would possibly also protect against reinfection, in the event of ongoing *Leishmania* exposure. For most HIV-associated opportunistic infections, prolonged prophylaxis or maintenance therapy has been found necessary to prevent reinfection and/or reactivation [66]. The recommended duration and/or CD4 threshold to discontinue preventive therapy will also have to be defined. However, there are currently no studies detailing how the risk of VL due to *L. donovani* (in East Africa) relates to CD4 cell count values and stages of immune suppression.

## Conclusions and Perspectives

Preventive treatment strategies have been explored and successfully implemented for all major HIV-associated opportunistic infections, following carefully conducted observational and interventional studies [66]. Given the poor treatment response of VL in HIV co-infected patients, even with ART, such strategies merit exploration.

A number of research gaps need to be filled (Key Learning Points). First, prospective cohort studies will have to be conducted to allow developing prognostic models, quantifying the risk of disease progression in different risk groups and across CD4 cell count levels. Subsequently, interventional studies will be needed to evaluate prophylactic or pre-emptive treatment strategies, ideally relying on an oral (combination) regimen. Careful evaluation of tolerability will be required, since increased toxicity has been observed with several anti-leishmanial drugs used to treat VL in patients with HIV co-infection. Nonetheless, the drugs are likely to be better tolerated in patients with less advanced HIV infection. Adherence to oral VL drugs, which has been cumbersome in VL patients, might be even more problematic in asymptomatic individuals. Based on the risk–benefit data, VL risk cut-offs will have to be identified at the local level to target treatment to those most likely to benefit.

A screen-and-treat strategy should be complemented by early ART initiation, as now recommended by WHO [79]. The decline in incidence of VL–HIV co-infection in Europe following the introduction of ART is a powerful testimony of the effectiveness of that strategy. However, in many settings, HIV is still detected at an advanced stage of immunosuppression, meaning individuals will remain at risk for an extended period before sufficient immune recovery has occurred. Additional efforts engendering early HIV detection are warranted.

Besides improved diagnosis and treatment of diseased individuals, a screen-and-treat approach to prevent VL could be a complementary strategy to decrease the burden of VL–HIV co-infection. In the process, issues like ARV drug interactions [80] and the potential emergence of drug resistant parasites will have to be evaluated, besides cost-effectiveness assessments. Since preventing the onset of VL also prevents the potential downstream consequences—including multiple relapses—targeted preventive efforts might be highly cost-effective. Other strategies to prevent HIV and *Leishmania* infection should be considered in parallel.

In conclusion, although a screen-and-treat approach strategy is conceptually attractive for resource-constrained settings with a high VL–HIV burden, the evidence base is currently lacking. Carefully conducted studies will be required to define its safety, feasibility and effectiveness. Such a strategy might also be valuable for other immunosuppressive conditions, currently most prevalent in high-income countries.

**Key Learning Points**

- Management of VL in HIV co-infection is complicated with high case fatality and relapse, warranting preventive strategies.
- A screen-and-treat approach targeting latent or the early stage of infection has successfully been implemented in other HIV-associated opportunistic infections.
- The basic understanding and evidence underpinning such an approach against VL in HIV coinfection is currently lacking; there are no validated markers for asymptomatic infection.
- Prospective cohort studies are required to quantify the risk of VL in different risk groups and across CD4 cell count levels.
- Interventional studies are needed to evaluate prophylactic or pre-emptive treatment strategies for those at risk, ideally relying on an oral (combination) regimen.

**Top Five Papers**

1. Hurissa Z, Gebre-Silassie S, Hailu W, Tefera T, Lalloo DG, et al. (2010) Clinical characteristics and treatment outcome of patients with visceral leishmaniasis and HIV co-infection in northwest Ethiopia. Trop Med Int Health 15: 848–855.
2. ter Horst R, Collin SM, Ritmeijer K, Bogale A, Davidson RN (2008) Concordant HIV infection and visceral leishmaniasis in Ethiopia: The influence of antiretroviral treatment and other factors on outcome. Clin Infect Dis 46: 1702–1709.
3. Rajasingham R, Meya DB, Boulware DR (2012) Integrating cryptococcal antigen screening and pre-emptive treatment into routine HIV care. J Acquir Immune Defic Syndr 59: e85–e91.
4. Hailu A, Gramiccia M, Kager PA (2009) Visceral leishmaniasis in Aba-Roba, south-western Ethiopia: Prevalence and incidence of active and subclinical infections. Ann Trop Med Parasitol 103: 659–670.
5. García-García JA, Martín-Sánchez J, Gállego M, Rivero-Román A, Camacho A, et al. (2006) Use of noninvasive markers to detect Leishmania infection in asymptomatic human immunodeficiency virus-infected patients. J Clin Microbiol 44: 4455-4458.
